# 

**Published:** 1799-11

**Authors:** 


NEW MEDICAL PUBLICATIONS IN OCTOBER.

Memoirs of the Medical Society of London, Vol. V. confifting of original Papers, and particular Cafes of Difeafe; alfo the Natural Hiftory of human inteftinal Worms; illuftrated with Plates. 9s. 6d. boards

Johnson.

Experiments with the Metallic Tractors in various Difeafes, as publifhed by Surgeons HERHOLDT and RASN, of the Royal Academy of Sciences, Copenhagen; tranflated into Englifh by Mr. C. KAMPFMULLER. Alfo, Reports of Cafes in England, demonftrating the efficacy of the Metallic Practice in complaints, both upon the human body, and on horfes, &c. by medical and other refpectable characters: edited by BENJAMIN D. PERKINS, A. M. 5s. boards. JOHNSON.

Hints on Temperance and Exercife; fhewing their advantages in the cure of diforders, and by which medicines are confiderably leffened; by J. TWEEDIE, Surgeon, &c. 2s. 6d. WILLIAMS.

The Anatomy of the Gravid Uterus, with practical inferences relative to Pregnancy and Labour; by JOHN BURNS, Surgeon in Glafgow. 8vo. 6s. boards. LONGMAN and REES.

An Effay on the Properties of Digitalis Purpurea, or Foxglove, by JOHN FERRIAR, M. D. 1s. 6d. CADELL and DAVIES.

---

### NEW PUBLICATIONS IN FRANCE.

*Clinique Chirurgicale, relative aux plaies, &c.* Clinical Surgery, as relating to wounds; intended as a Sequel to the Art of applying Bandages. By LOMBARD, Member of the National Inftitut. 8vo. (price 1 rix-dollar, 6 grofch, or about 5s.) *Strasburg.* Levrault.

*Tableau élémentaire de la féméiotique, &c.* Elements of Semiology; or, the Knowledge of the Signs of Difeafes. By BROUSONNET. 8vo. (12 grofch, or 2s.) *Ibid.*

*Elemens de Myologie, &c.* Elements of Myology and Syndefmology. By TH. LAUTH. Two vols. 8vo. (price 2 rix-dollars, or 8s.) *Ibid.*

*Elemens de la Médecine, Theoretique et Pratique, &c.* Elements of the Theory and Practice of Medicine; containing the general pathology, the epidemic conftitutions of Hippocrates, and their analyfis, the doctrine of prognoftics, and nofology. By TOURTELLE. Three vols. 8vo. (price 4 rix-dollars, or 16s.) *Ibid.*

---

### NEW PUBLICATION IN GERMANY.

*Uber den Einflufs des Medicinalwefens auf den Staat, &c.*—On the influence of medical affairs on the ftate; and on the neglected medical police in moft of the German ftates: by the Aulic Counfellor SCHÖPF, prefident of a medical college, &c. 8vo. (pr. 6 grofch or 1s.) 17[illegible]9.

---

### TO CORRESPONDENTS.

*We acknowledge the receipt of communications from Meffrs. Spry, Fowler, Ayrton, Davies, Rowlands, Cuftance, Mills, Huggan, Reeve, Thackeray, T. P. Graham, F. A. Chriftie, Franks, Dr. Vaughan, &c. which fhall be noticed without delay.*

*The letter, signed S. N. dated Oct.* 9, 1799, *cannot, for obvious reasons, be inserted; for although it contain perhaps a fatisfactory anfwer to another letter marked with initials, yet it would be inconfiftent with our plan, to extend* anonymous *disputes.*

